# Supplementary material for: Automated extracellular volume fraction measurement for diagnosis and prognostication in patients with light-chain cardiac amyloidosis
Source: PLoS One. 2025 Jan 22;20(1):e0317741. doi: 10.1371/journal.pone.0317741 (PMC11753688; doi:10.1371/journal.pone.0317741)
Supplement: S1 Fig — (PDF) [file pone.0317741.s002.pdf]

S1 Fig. Schematic figure of the native T1 and ECV measurements derived from AI-automated segmentation T1 mapping

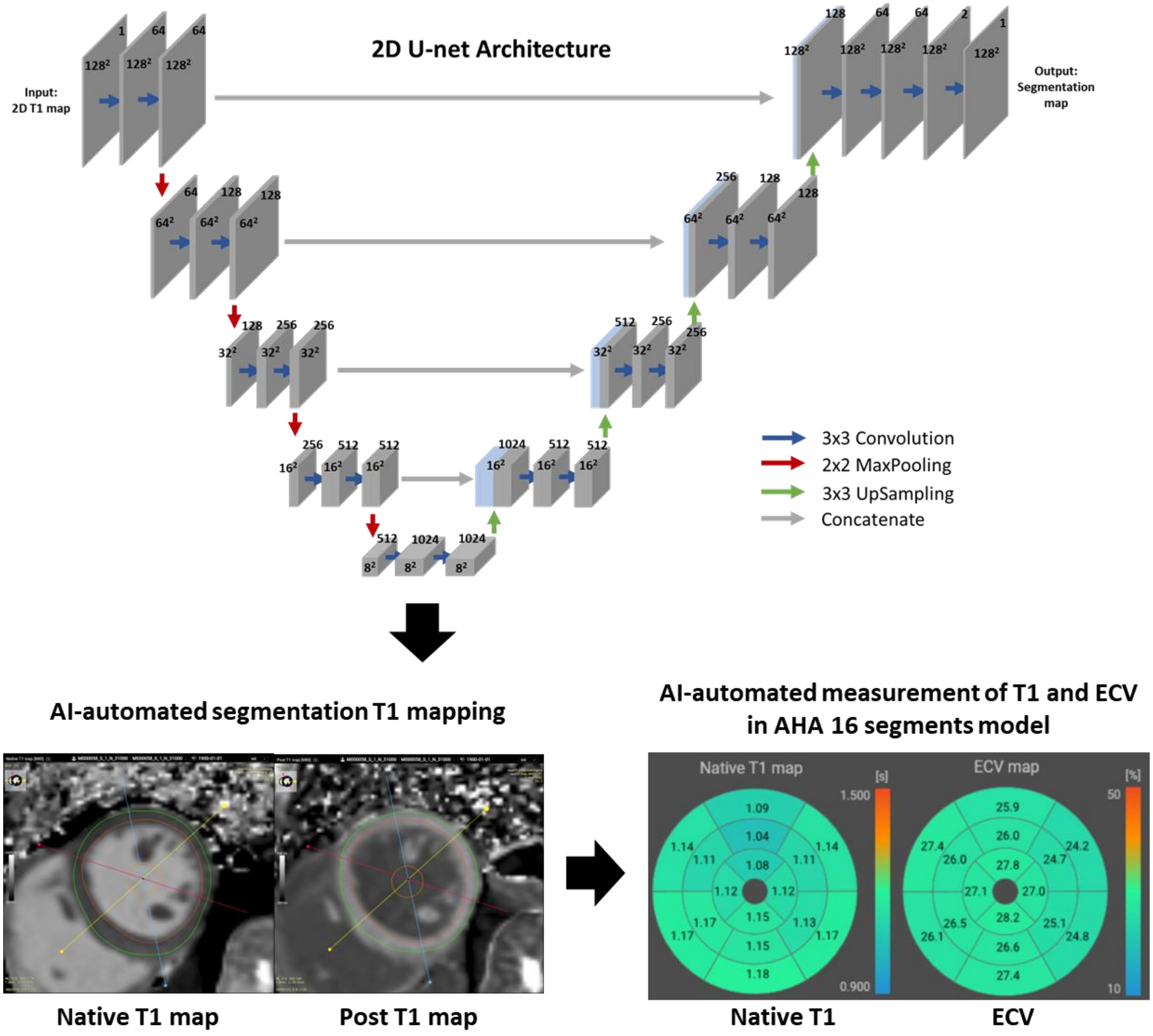

Abbreviations: AI, artificial intelligence; ECV, extracellular volume fraction; AHA, American Heart Association
