# Supplementary material for: Automated extracellular volume fraction measurement for diagnosis and prognostication in patients with light-chain cardiac amyloidosis
Source: PLoS One. 2025 Jan 22;20(1):e0317741. doi: 10.1371/journal.pone.0317741 (PMC11753688; doi:10.1371/journal.pone.0317741)
Supplement: S2 Fig — (PDF) [file pone.0317741.s003.pdf]

S2 Fig. Accuracy of automated native T1 and ECV measurements

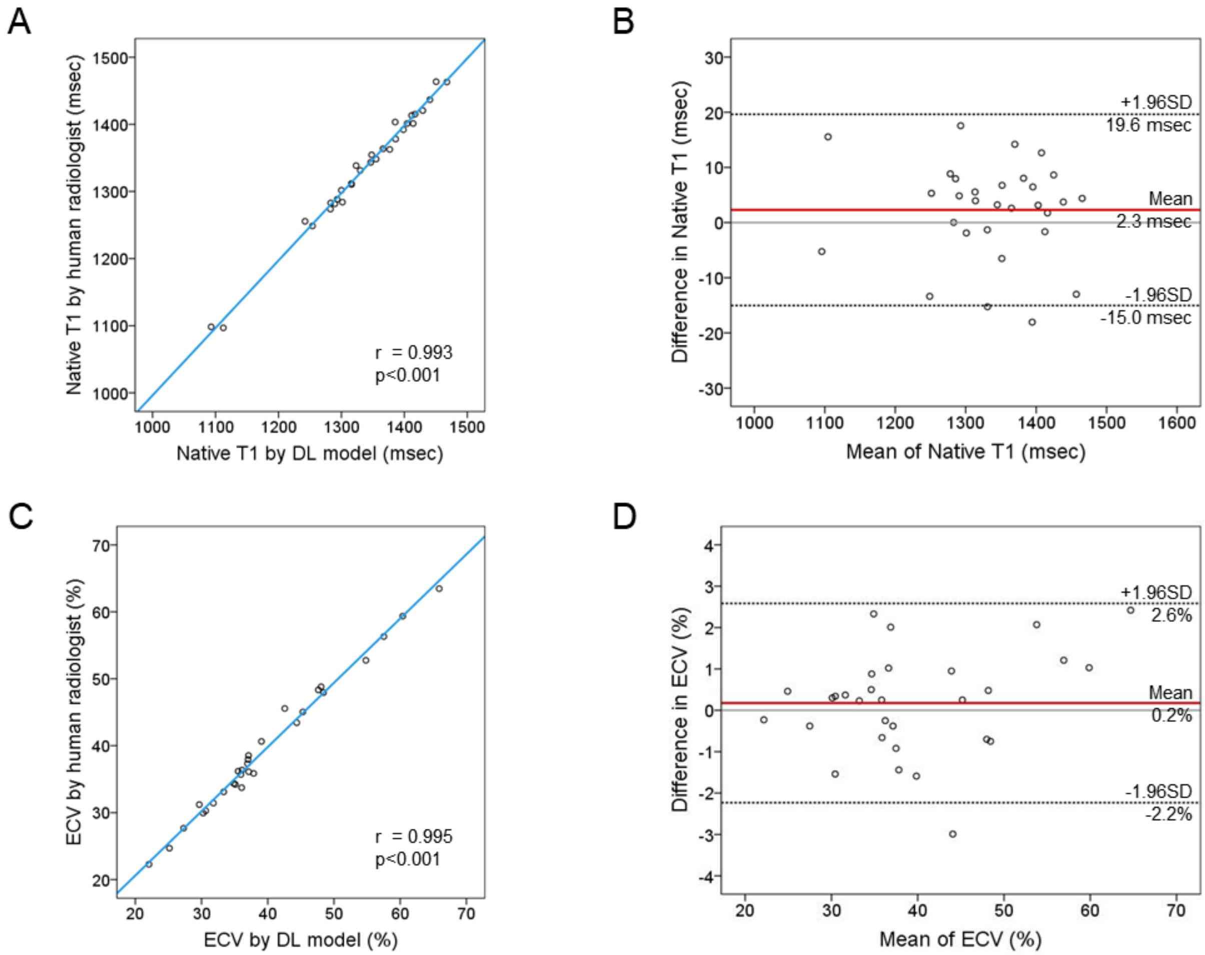

Correlations and agreements between the automated DL-based vs. manual (human radiologist) native T1 and ECV measurements are shown.

Abbreviations: DL, deep learning; SD, standard deviation; ECV, extracellular volume fraction
