# Supplementary material for: Automated extracellular volume fraction measurement for diagnosis and prognostication in patients with light-chain cardiac amyloidosis
Source: PLoS One. 2025 Jan 22;20(1):e0317741. doi: 10.1371/journal.pone.0317741 (PMC11753688; doi:10.1371/journal.pone.0317741)
Supplement: S3 Fig — (PDF) [file pone.0317741.s004.pdf]

S3 Fig. ROC curves for the prediction of clinical outcomes of AL-CA using the automated native T1 and ECV measurements

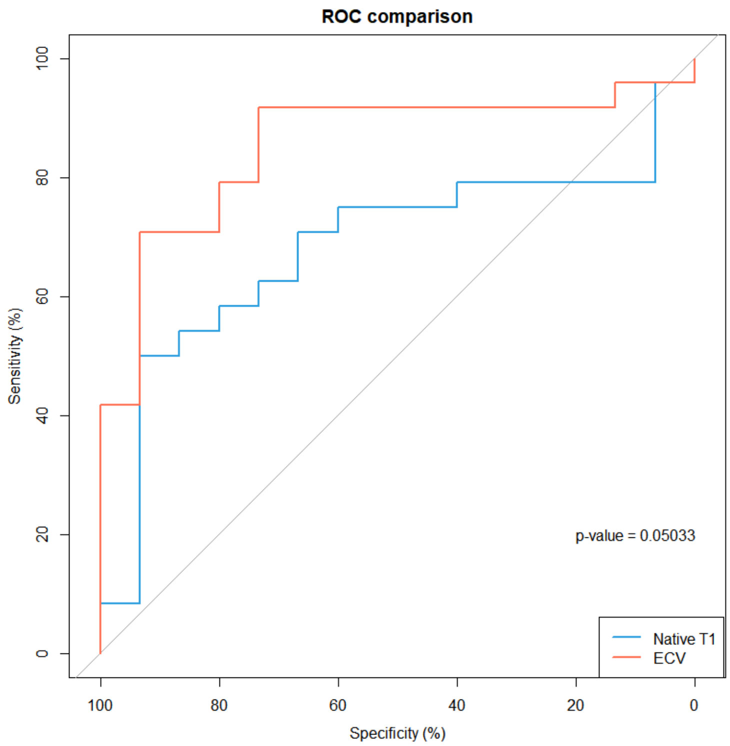

The performance of the AI-assisted automated native T1 and ECV measurements in the prediction of cardiovascular death and hospitalization for heart failure among patients with AL-CA is shown in the ROC curves.

Abbreviations: ROC, receiver operating characteristic; AI, artificial intelligence; ECV, extracellular volume fraction; AUC, area under the curve; CA, cardiac amyloidosis; LVH, left ventricular hypertrophy.
