# Supplementary material for: Automated extracellular volume fraction measurement for diagnosis and prognostication in patients with light-chain cardiac amyloidosis
Source: PLoS One. 2025 Jan 22;20(1):e0317741. doi: 10.1371/journal.pone.0317741 (PMC11753688; doi:10.1371/journal.pone.0317741)
Supplement: S4 Fig — (PDF) [file pone.0317741.s005.pdf]

S4 Fig. Event-free survival of patients with TTR-CA according to the automated ECV

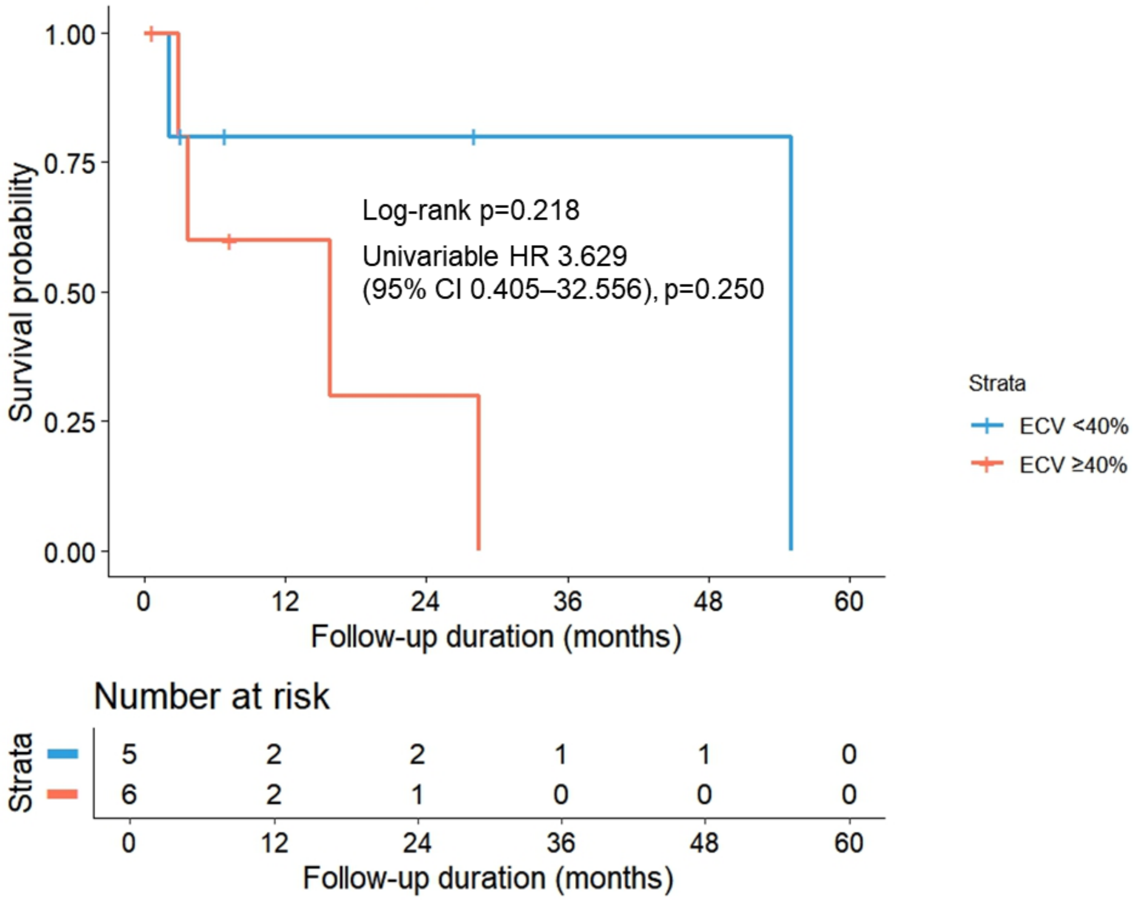

Clinical outcomes (composite of cardiovascular death and hospitalization for heart failure) were compared between the subgroups divided by automated ECV (40%).

Abbreviations: HR, hazard ratio; ECV, extracellular volume fraction; AUC, area under the curve; TTR-CA, transthyretin cardiac amyloidosis.
