# Supplementary figures and images for: Automated extracellular volume fraction measurement for diagnosis and prognostication in patients with light-chain cardiac amyloidosis
Source: PLoS One. 2025 Jan 22;20(1):e0317741. doi: 10.1371/journal.pone.0317741 (PMC11753688; doi:10.1371/journal.pone.0317741)

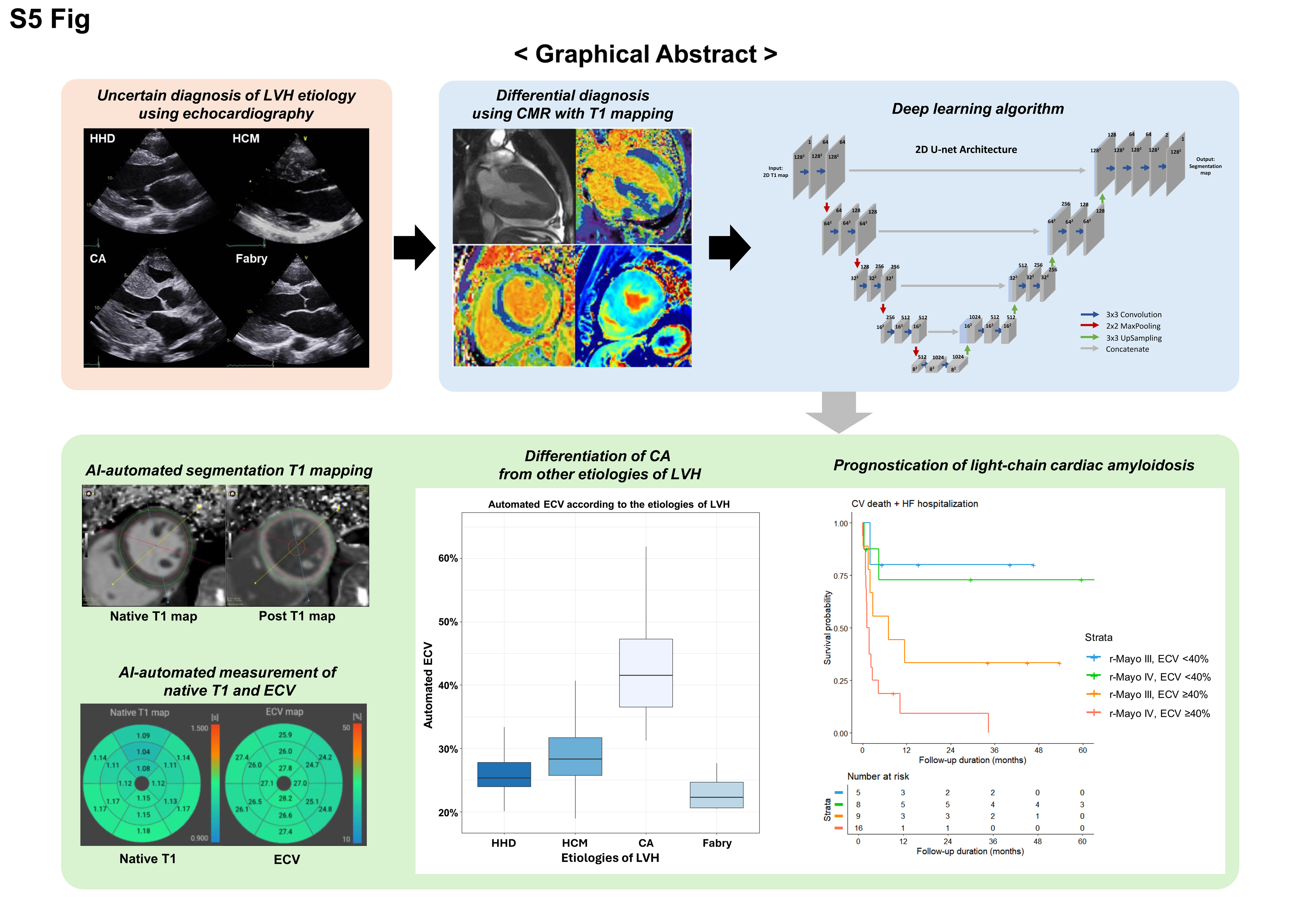

Supplement: S5 Fig — Abbreviations: ECV, extracellular volume fraction; AL-CA, AL cardiac amyloidosis; CV, cardiovascular; HF, heart failure; r-Mayo, revised Mayo staging. (TIF) [file pone.0317741.s006.tif]
