# Supplementary material for: Automated extracellular volume fraction measurement for diagnosis and prognostication in patients with light-chain cardiac amyloidosis
Source: PLoS One. 2025 Jan 22;20(1):e0317741. doi: 10.1371/journal.pone.0317741 (PMC11753688; doi:10.1371/journal.pone.0317741)
Supplement: S1 Table — (PDF) [file pone.0317741.s007.pdf]

S1 Table. Univariable predictors of the composite outcome among patients with AL-CA

	**Unadjusted HR (95% CI)**	**P-value**
**Age (per +1 year)**	0.953 (0.910 – 0.999)	0.044
**Age ≥60 years**	0.100 (0.035 – 0.288)	<0.001
**Age ≥65 years**	0.529 (0.225 – 1.245)	0.145
**Age ≥70 years**	0.768 (0.344 – 1.713)	0.519
**Male sex**	1.012 (0.440 – 2.328)	0.978
**Hypertension**	0.627 (0.249 – 1.583)	0.323
**Diabetes mellitus**	0.702 (0.262 – 1.881)	0.481
**Chronic kidney disease**	0.771 (0.319 – 1.863)	0.564
**Body-mass index (per +1 kg/m^2)**	0.914 (0.783 – 1.067)	0.253
**Systolic BP (per +1 mmHg)**	0.967 (0.944 – 0.991)	0.007
**Systolic BP <100 mmHg**	1.996 (0.844 – 4.720)	0.116
**Systolic BP <110 mmHg**	3.114 (1.359 – 7.133)	0.007
**Diastolic BP (per +1 mmHg)**	0.981 (0.949 – 1.015)	0.266
**Hemoglobin (per +1 g/dL)**	1.075 (0.809 – 1.428)	0.619
**Hematocrit (per +1%)**	1.016 (0.920 – 1.121)	0.756
**Glomerular filtration rate (per +1 mL/min/1.73m^2)**	1.007 (0.990 – 1.025)	0.422
**Albumin (per +1 g/dL)**	0.661 (0.303 – 1.443)	0.299
**Delta FLC (per +1)**	1.000 (0.999 – 1.001)	0.615
**Delta FLC ≥18 mg/dL**	1.517 (0.566 – 4.066)	0.408
**NT-proBNP (per +1 pg/mL)**	1.000 (1.000 – 1.000)	0.073
**NT-proBNP ≥1800 pg/mL**	1.885 (0.442 – 8.046)	0.392
**CKMB (per +1 ng/mL)**	0.996 (0.871 – 1.138)	0.947
**Troponin I (per +1 ng/mL)**	0.999 (0.591 – 1.688)	0.997
**Troponin T (per +1 ng/mL)**	235.334 (2.722 – 20348.545)	0.016
**Troponin I ≥0.1 ng/mL**	1.593 (0.593 – 4.282)	0.356
**Troponin T ≥0.025 ng/mL**	0.433 (0.146 – 1.286)	0.132
**Elevated troponin***	N/A	
**Native T1 (per +1 msec)**	1.007 (1.001 – 1.012)	0.021
**Post T1 (per +1 msec)**	0.994 (0.988 – 0.999)	0.029
**ECV (per +1%)**	1.097 (1.045 – 1.152)	<0.001
**T2 (per 1 msec)**	1.041 (1.003 – 1.080)	0.033

**LV-EDV (per +1 mL)**	1.005 (0.983 – 1.027)	0.662
**LV-EF (per +1%)**	0.956 (0.912 – 1.002)	0.061
**LV-EF <50%**	1.454 (0.538 – 3.931)	0.461
**LV-EF <55%**	2.277 (1.012 – 5.122)	0.047
**LV-EF <60%**	0.999 (0.437 – 2.285)	0.998
**LV-MI (per +1 g/m^2)**	1.005 (0.993 – 1.017)	0.381
**LAVI (per +1 mL/m^2)**	0.999 (0.973 – 1.026)	0.946
**E/e' (per +1)**	1.008 (0.966 – 1.052)	0.722
**TR Vmax (per +1 m/sec)**	1.008 (0.967 – 1.051)	0.703
**LV-GLS (per +1%)**	0.832 (0.724 – 0.955)	0.009
**LV-GLS <8%**	2.484 (1.104 – 5.591)	0.028
**LV-GLS <10%**	2.632 (1.039 – 6.666)	0.041
**LV-GLS <12%**	3.269 (0.764 – 13.976)	0.110
**Revised Mayo stage (IV vs. III)**	1.846 (0.764 – 4.465)	0.173
**Presence of LGE**	0.608 (0.081 – 4.581)	0.629
**Diffuse distribution of LGE**	1.566 (0.674 – 3.638)	0.297
**Subendocardial ring-pattern of LGE**	4.105 (0.553 – 30.486)	0.167
**ECV ≥40%**	6.470 (1.910 – 21.914)	0.003
**ECV ≥45%**	4.214 (1.789 – 9.926)	0.001

* Elevated troponin was defined as either troponin T ≥0.025 ng/mL or troponin I ≥0.1 ng/mL.

Abbreviations: HR, hazard ratio; CI, confidence interval; BP, blood pressure; FLC, free light chain; NT-proBNP, N-terminal proB-type natriuretic peptide; CKMB, creatine kinase-myocardial band; ECV, extracellular volume fraction; LV, left ventricular; EDV, end-diastolic volume; EF, ejection fraction; MI, mass index; LAVI, left atrial volume index; TR, tricuspid regurgitation; GLS, global longitudinal strain; N/A, not applicable.
