# Supplementary material for: Automated extracellular volume fraction measurement for diagnosis and prognostication in patients with light-chain cardiac amyloidosis
Source: PLoS One. 2025 Jan 22;20(1):e0317741. doi: 10.1371/journal.pone.0317741 (PMC11753688; doi:10.1371/journal.pone.0317741)
Supplement: Attachment — Submitted filename: Response letter_PLOS One_R1.docx [file pone.0317741.s008.docx]

December 1^st^, 2024

Emily Chenette, PhD
Editor-in-Chief

*PLOS ONE*

Giuseppe Limongelli, MD, PhD
Academic Editor
*PLOS ONE*

Manuscript ID#: **PONE-D-24-22784 (*Minor revision*)**

Manuscript title: **Automated extracellular volume fraction measurement for diagnosis and prognostication in patients with light-chain cardiac amyloidosis**

Dear Editor:

On behalf of all the authors, I would like to thank you and the reviewers of *PLOS ONE* for taking the time and effort to review our manuscript. The thoughtful comments and scientific questions raised during the revision process provided valuable insights into our study and greatly strengthened our manuscript. The revised portions of the text are highlighted in the manuscript.

We hope that the revised version will better meet the requirements of *PLOS ONE* for publication. We thank you again for your constructive and detailed review and hope to hear good news in the near future.

Best regards,

In-Chang Hwang, MD

Associate Professor

Department of Internal Medicine, Seoul National University College of Medicine

Cardiovascular Center, Seoul National University Bundang Hospital

82 Gumi-ro-173-gil, Bundang, Seongnam, Gyeonggi, 13620, South Korea

Tel.: +82-31-787-7065 / Fax: +82-31-787-4290

E-mail: [inchang.hwang@gmail.com](mailto:inchang.hwang@gmail.com)

Eun Ju Chun, MD, PhD

Professor,

Department of Radiology, Seoul National University College of Medicine,

Department of Radiology, Cardiovascular Center, Seoul National University Bundang Hospital, 82 Gumi-ro-173-gil, Bundang, Seongnam, Gyeonggi 13620, South Korea

Tel.: +82-31-787-7618

Fax: +82-31-787-4011

E-mail: [humandr@snubh.org](mailto:humandr@snubh.org)

***Point-by-point Responses to Reviewer #1 Comments to Author:***

**General comment**

The authors present an interesting study on the ability of AI automated segmentation T1 mapping to detect cardiac amyloidosis (CA) in patients with LVH. Also, they show that automated ECV measurements in patients with AL-CA improve prognostication of the disease.

**Response to the general comment**

We thank the reviewer’s considerate review and comments. We believe that the revision processes have further improved the strengths of our study. We tried our best to clarify the raised comments, and if the issues were not able to be addressed within the present study, we honestly acknowledged the limitations of our study, in order to provide appropriate background information for the readers. We just hope that the revised version with responses to the reviewer can better comply with the publication standards of *PLOS ONE*.

**Comment #1**

It would be useful to clarify few points: were the patients with suspicion of LVH consecutive patients referred for MRI due to LVH ? If not, why?

**Response to comment #1**

We sincerely thank the reviewer for this valuable comment. In the present study, we identified consecutive patients who underwent cardiac MRI for differential diagnosis of the etiology, between 2011 and 2023. In the revised manuscript, we indicated that the study population was retrospectively identified on a consecutive manner.

**Changes in accordance with comment #1**

- **Abstract (page 2):** A total of 300 consecutive patients who underwent CMR for differential diagnosis of LVH were analyzed.
- **Methods – Study design and cohort (page 4):** We retrospectively identified 300 consecutive patients (47 with HHD, 198 with HCM, 50 with CA [39 with AL-CA, and 11 with TTR-CA], and 5 with Fabry disease [FD]) (**Fig 1**) who underwent CMR for differential diagnosis of the etiology, based on the detection of LVH on echocardiography, between 2011 and 2023.

**Comment #2**

Why was prognostic value of ECV calculated only for AL patients and not for ATTR as well?

**Response to comment #2**

Thank you for your valuable feedback. Among the total 300 consecutive patients who underwent CMR for differential diagnosis of LVH, we have found 50 patients with cardiac amyloidosis, consisting of 39 patients with AL-CA and 11 patients with TTR-CA. Those 11 patients with TTR-CA were further divided according to the subtype of TTR: 3 patients with hereditary TTR-CA (genetic; hATTR) and 8 patients with wild-type TTR-CA (senile; wtATTR). Given the number of patients in each ATTR subgroup (hATTR and wtATTR), we performed survival analysis among the total 11 patients with TTR-CA according to the ECV. Although we could not obtain statistically significant results due to the small number of patients, it was observed that the patients with TTR-CA and higher ECV levels (automated ECV ≥40%) tended to have higher risk of composite outcomes (cardiovascular death and heart failure hospitalization) compared to those with lower ECV levels (<40%).

In the revised manuscript, we added these findings as Supplementary results and provided interpretations in the Limitation section, as below:

**Changes in accordance with comment #2**

- **Results (page 16): Prognostic value of ECV in patients with TTR.** The prognostic value of the automated ECV was further assessed in patients with TTR-CA. Among the 11 patients with TTR-CA included in the present study (3 with hereditary TTR-CA [genetic; hATTR] and 8 with wild-type TTR-CA [senile; wtATTR]), 1 patient died of cardiovascular cause and 6 patients experienced hospitalization for heart failure. The AUC values for the study outcome using the automated native T1 and ECV were 0.600 (95% CI 0.226–0.974; p=0.584) and 0.700 (95% CI 0.376–1.000; p=0.273), respectively. These patients with TTR-CA were dichotomized according to the median value of automated ECV (40%), and the prognosis was compared between those with ECV <40% and those with ECV ≥40% (**S4 Fig.**). Although we could not obtain statistically significant results due to the small number of patients, it was observed that the patients with TTR-CA and higher ECV levels (automated ECV ≥40%) tended to have higher risk of composite outcomes (cardiovascular death and heart failure hospitalization) compared to those with lower ECV levels (<40%) (Log-rank p=0.218; unadjusted HR 3.629, 95% CI 0.405–32.556, p=0.250).
- **S4 Fig. Event-free survival of patients with TTR-CA according to the automated ECV**

*Clinical outcomes (composite of cardiovascular death and hospitalization for heart failure) were compared between the subgroups divided by automated ECV (40%).*

*Abbreviations: HR, hazard ratio; ECV, extracellular volume fraction; AUC, area under the curve; TTR-CA, transthyretin cardiac amyloidosis.*

- **Limitation (page 21):** Second, the prognostic value of T1 mapping parameters was assessed only among the patients with AL-CA, but not for those with other etiologies. We observed a trend toward worse prognosis in patients with TTR-CA and higher ECV (automated ECV ≥40%) compared to those with lower ECV; however, the number of TTR-CA cases in this study was limited to 11, necessitating future studies for statistically meaningful analysis. Patients with HCM and HHD did not experience sufficient number of events, and the number of patients with FD was too small for further analysis.

***Point-by-point Responses to Reviewer #2 Comments to Author:***

**General comment**

The authors present a study on the use of an artificial intelligence (AI) -based T1 mapping in 300 patients diagnosed with left ventricular hypertrophy (LVH). CMR AI-based T1-mapping was able to detect cardiac amyloidosis (CA) and provided prognostic information in those with AL-CA.

The potential use of AI based T1-mapping and automated extracellular volume (ECV) calculation in diagnosis and prognostication of LVH is clinically relevant.

**Response to the general comment**

We appreciate the reviewer’s thoughtful comments and feedback. We believe that the revision process has helped enhance the strengths of our study. We have made every effort to address the comments raised, and where certain issues could not be fully resolved within the scope of this study, we have acknowledged these limitations to provide readers with a clear context. We hope that the revised version now aligns more closely with the publication standards of *PLOS ONE*.

**Comment #1**

While I understand quantitative assessment of LGE was not available to the authors, it would be useful to include how many patients presented with LGE and the distribution in the myocardium.

**Comment #2**

Also, CMR-derived LVEF and LVEDV measures should be included in Table 1.

**Response to comments #1 and #2**

We sincerely thank the reviewer for this valuable comment. According to the reviewer’s advice, we have added the assessment of LGE, as well as LV volumetric measures. The LV-EDV was the smallest in patients with AL-CA (74.0±24.9 mL), followed by those with TTR-CA (81.2±24.4 mL), and was the largest in patients with Fabry disease (107.7±57.8 mL). LV-EF was similar across the subgroups of HHD, HCM, and Fabry disease, but was significantly lower in patients with AL-CA (59.0±11.0%).

It was noted that LGE was observed in most of the patients with HCM (97.0%), AL-CA (97.4%), TTR-CA (100.0%), and Fabry disease (80.0%), whereas 40% of patients with HHD had LGE, which mainly showed focally-distributed patchy LGE. Typically, patients with AL-CA and TTR-CA showed diffuse distribution of LGE with subendocardial ring-type enhancement, and patients with HCM showed focal or multifocal distribution of LGE with patchy segmental enhancement. It was noted that the extent and distribution pattern of LGE were not associated with study outcomes, because of the typically similar LGE patterns observed in patients with AL-CA (35 patients [89.7%] of AL-CA had diffuse LGE with subendocardial ring-type distribution).

In the revised manuscript, we added descriptions regarding the LV volumetric measures and LGE assessment findings in the Methods section, Results section, Limitations section, and **Table 1**, as below.

**Changes in accordance with comments #1 and #2**

- **Methods (pages 6 – 7):** LGE extent was categorized into focal (localized distribution), multifocal (distribution across several locations), and diffuse (involving at least three contiguous myocardial segments). Additionally, the distribution patterns were classified into six distinct types and analyzed as follows; patchy segmental, transmural, mid-layer, RV insertion point, subendocardial ring, and nonspecific.
- **Results (page 9):** The mean LVMI was 118.7±33.7 g/m^2^ in patients with HHD, 134.9±32.7 g/m^2^ in those with HCM, 134.0±36.1 g/m^2^ in those with AL-CA, 143.0±54.0 g/m^2^ in those with TTR-CA, and 162.2±80.2 g/m^2^ in those with FD. LV-EDV measured by CMR was smallest in patients with AL-CA (74.0±24.9 mL), followed by those with TTR-CA (81.2±24.4 mL), and largest in patients with Fabry disease (107.7±57.8 mL). LV-EF measured by CMR was comparable among patients with HHD, HCM, and Fabry disease but was significantly lower in those with AL-CA (59.0±11.0%). LGE was observed in the majority of patients with HCM (97.0%), AL-CA (97.4%), TTR-CA (100.0%), and Fabry disease (80.0%). In contrast, only 40% of patients with HHD showed LGE, which primarily presented as focal, patchy enhancement. Patients with AL-CA and TTR-CA typically exhibited diffuse LGE with a subendocardial ring-like pattern, while those with HCM demonstrated focal or multifocal LGE with patchy, segmental enhancement. Patients with CA had the highest native T1 values (1444.6±83.4 msec in AL-CA, and 1418.3±72.8 msec in TTR-CA), followed by patients with HCM (1319.4±56.9 msec), and those with HHD (1291.4±38.9 msec), and patients with FD (1143.9±80.3 msec) (**Fig 3A**).
- **Results (page 13):** The extent and distribution pattern of LGE were not associated with the study outcomes due to the predominantly similar LGE patterns observed in patients with AL-CA: specifically, 35 patients (89.7%) with AL-CA exhibited diffuse LGE with a characteristic subendocardial ring-type distribution (**Table 1** and **S1 Table**).
- **Limitations (page 21):** Third, while the qualitative assessment of LGE, including its distribution and pattern, proved useful for differentiating LVH etiologies in our study population, it was not employed for prognostication due to the typically similar LGE patterns observed in patients with AL-CA. Additionally, we could not provide a quantitative assessment of LGE in this study. Instead, we focused on the clinical usefulness of the automated T1 mapping parameters by DL algorithm. Nonetheless, we acknowledge that future studies on the radiomics features of LGE in the automated CMR interpretation are warranted.

**Revised Table 1. Baseline characteristics**

|  | **HHD**  **(n=47)** | **HCM**  **(n=198)** | **CA (n=50)** | | **Fabry disease**  **(n=5)** | **p-value** |
| --- | --- | --- | --- | --- | --- | --- |
|  |  |  | **AL-CA (n=39)** | **TTR-CA (n=11)** |  |  |
| **CMR measurements** |  |  |  |  |  |  |
| **LV-EDV by CMR, mL** | 85.7±23.4 | 87.8±28.2 | 74.0±24.9 | 81.2±24.4 | 107.7±57.8 | 0.022 |
| **LV-ESV by CMR, mL** | 30.5±18.1 | 29.7±16.8 | 31.1±16.2 | 31.0±11.5 | 38.6±26.8 | 0.816 |
| **LV-EF by CMR, %** | 66.2±12.7 | 67.3±10.6 | 59.0±11.0 | 61.2±11.5 | 65.2±5.9 | 0.001 |
| **Presence of LGE** | 19 (40.4%) | 192 (97.0%) | 38 (97.4%) | 11 (100.0%) | 4 (80.0%) | <0.001 |
| **Extent of LGE** |  |  |  |  |  | <0.001 |
| **- Focal** | 14 (29.8%) | 77 (38.9%) | 2 (5.1%) | 0 (0.0%) | 2 (40.0%) |  |
| **- Multifocal** | 3 (6.4%) | 104 (52.5%) | 1 (2.6%) | 1 (9.1%) | 2 (40.0%) |  |
| **- Diffuse** | 2 (4.3%) | 11 (5.6%) | 35 (89.7%) | 10 (90.9%) | 0 (0.0%) |  |
| **Distribution pattern of LGE** |  |  |  |  |  |  |
| **- Patchy segmental** | 8 (17.0%) | 122 (61.6%) | 2 (5.1%) | 0 (0.0%) | 3 (60.0%) | <0.001 |
| **- Transmural** | 0 (0.0%) | 7 (3.5%) | 6 (15.4%) | 2 (18.2%) | 0 (0.0%) | 0.002 |
| **- Mid-layer** | 0 (0.0%) | 2 (1.0%) | 0 (0.0%) | 0 (0.0%) | 0 (0.0%) | 0.904 |
| **- RV insertion point** | 9 (19.1%) | 70 (35.4%) | 1 (2.6%) | 0 (0.0%) | 0 (0.0%) | <0.001 |
| **- Subendocardial ring** | 1 (2.1%) | 7 (3.5%) | 35 (89.7%) | 11 (100.0%) | 1 (20.0%) | <0.001 |
| **- Nonspecific** | 1 (2.1%) | 0 (0.0%) | 0 (0.0%) | 0 (0.0%) | 0 (0.0%) | 0.249 |
| **Native T1, msec** | 1291.4±38.9 | 1319.4±56.9 | 1444.6±83.4 | 1418.3±72.8 | 1143.9±80.3 | <0.001 |
| **Post T1, msec** | 486.1±47.2 | 451.2±71.9 | 397.3±79.9 | 365.3±73.9 | 496.9±57.1 | <0.001 |
| **Post-contrast T2, msec** | 49.6±4.0 | 49.5±3.1 | 61.6±11.6 | 52.3±2.8 | 46.8±4.8 | <0.001 |
| **ECV, %** | 26.1±3.3 | 29.5±5.9 | 43.6±8.1 | 40.0±4.3 | 23.2±3.0 | <0.001 |

**Revised S1 Table. Univariable predictors of the composite outcome among patients with AL-CA**

|  | **Unadjusted HR (95% CI)** | **P-value** |
| --- | --- | --- |
| **Presence of LGE** | 0.608 (0.081 – 4.581) | 0.629 |
| **Diffuse distribution of LGE** | 1.566 (0.674 – 3.638) | 0.297 |
| **Subendocardial ring-pattern of LGE** | 4.105 (0.553 – 30.486) | 0.167 |
| **ECV ≥40%** | 6.470 (1.910 – 21.914) | 0.003 |
| **ECV ≥45%** | 4.214 (1.789 – 9.926) | 0.001 |

**Comment #3**

Finally, it should be highlighted in the limitations that there is no testing/validation cohort and the results might not be generalisable.

**Response to comment #3**

We agree with the reviewer’s valuable comment. According to the reviewer’s advice, we added in the limitation that, because of the lack of testing/validation cohorts, our findings should be interpreted with caution and might not be generalizable in other study populations or different clinical settings.

**Changes in accordance with comment #3**

- **Limitations (page 21):** Finally, due to the absence of a testing or validation cohort in this study, our findings should be interpreted with caution and warrant prospective validation in terms of improving the diagnostic process and prognosis.

***Point-by-point Responses to the Journal Requirements:***

**Item #1**

When submitting your revision, we need you to address these additional requirements.

Please ensure that your manuscript meets PLOS ONE's style requirements, including those for file naming. The PLOS ONE style templates can be found at 
<https://journals.plos.org/plosone/s/file?id=wjVg/PLOSOne_formatting_sample_main_body.pdf> and 
<https://journals.plos.org/plosone/s/file?id=ba62/PLOSOne_formatting_sample_title_authors_affiliations.pdf>

**Response to Item #1**

In the revised manuscript, we followed the PLOS ONE’s style requirements.

**Item #2**

Please note that PLOS ONE has specific guidelines on code sharing for submissions in which author-generated code underpins the findings in the manuscript. In these cases, all author-generated code must be made available without restrictions upon publication of the work. Please review our guidelines at <https://journals.plos.org/plosone/s/materials-and-software-sharing#loc-sharing-code> and ensure that your code is shared in a way that follows best practice and facilitates reproducibility and reuse.

**Response to Item #2**

According to the PLOS ONE’s guidelines on code sharing, we provided the codes for statistical analyses in the revised manuscript files (https://github.com/Inchang-Hwang/CMR-ECV-for-AL-CA). Regarding the automated measurements of native T1 and ECV on cardiac MR images, we believe that the deep learning algorithm for automatically measuring native T1 and ECV in cardiac MRI images is not directly relevant to the content of this study, as it is incorporated into an analysis program whose accuracy has already been validated in multiple prior studies (Ref: Korean J Radiol. 2022;23(12):1251-9 / Korean J Radiol. 2023;24(5):395-405). Additionally, as this analysis program, which includes the deep learning algorithm, is commercialized by Phantomics Inc., we ask for your understanding that specific details cannot be disclosed due to company confidentiality.

Below is the revised Data Availability Statement.

- **Data availability statement:** Data used in this study cannot be made publicly available because of the strict ethical restrictions set by the IRB of Seoul National University Bundang Hospital (https://e-irb.snubh.org). Please contact the corresponding authors (inchang.hwang@gmail.com or humandr@snubh.org) or the ethics board at SNUBH (snubhirb@gmail.com) for further inquiries regarding data availability within the scope permitted by the IRB. The code for statistical analysis utilized in the present study was released (https://github.com/Inchang-Hwang/CMR-ECV-for-AL-CA).

**Item #3**

Thank you for stating in your Funding Statement: 
*This work was supported by the Medical AI Clinic Program through the National IT Industry Promotion Agency (NIPA) funded by the Ministry of Science and ICT (MSIT) of the Republic of Korea, and by research grants from Seoul National University Bundang Hospital (Grant No. 02-2017-0040 and 06-2020-0130).*

Please provide an amended statement that declares *all* the funding or sources of support (whether external or internal to your organization) received during this study, as detailed online in our guide for authors at <http://journals.plos.org/plosone/s/submit-now>. Please also include the statement “There was no additional external funding received for this study.” in your updated Funding Statement. 
Please include your amended Funding Statement within your cover letter. We will change the online submission form on your behalf.

**Response to Item #3**

We confirm that there was no additional external funding received for this study. According to the journal’s requirements, we amended Funding Statement, Competing Interests Statement, and Author Contributions Statement, as below:

- **Funding:** This work was supported by the Medical AI Clinic Program through the National IT Industry Promotion Agency (NIPA), funded by the Ministry of Science and ICT (MSIT) of the Republic of Korea, and by research grants from Seoul National University Bundang Hospital (Grant Nos. 02-2017-0040 and 06-2020-0130). Pan Ki Kim and Byoung Wook Choi are founders of Phantomics, Inc. (Seoul, Korea), which provided support for the software used in this study. Phantomics, Inc. also funded salaries for PKK and BWC but had no role in the study design, data collection, analysis, publication decisions, or manuscript preparation. The specific contributions of each author are detailed in the ‘author contributions’ section. No additional external funding was received for this study.
- **Competing Interests:** Pan Ki Kim and Byoung Wook Choi are founders of Phantomics, Inc. (Seoul, Korea), which provided support for the software used in this study. Phantomics, Inc. also funded salaries for PKK and BWC but had no influence on the study's design, data collection, analysis, publication decisions, or manuscript preparation. This statement does not alter the authors' adherence to PLOS ONE policies on data and material sharing. All other authors (ICH, EJC, MK, JP, HMC, YEY, and GYC) declare that they have no competing interests related to Phantomics, Inc. or any other funders or institutions, including any affiliations involving employment, consultancy, patents, products in development, or marketed products. The specific contributions of each author are detailed in the ‘author contributions’ section.
- **Author Contributions**
  - **Conceptualization:** In-Chang Hwang, Eun Ju Chun, Myeongju Kim, Jiesuck Park, Hong-Mi Choi, Yeonyee E. Yoon, Goo-Yeong Cho.
  - **Data curation:** In-Chang Hwang, Eun Ju Chun.
  - **Formal analysis:** In-Chang Hwang.
  - **Funding acquisition:** In-Chang Hwang, Eun Ju Chun.
  - **Investigation:** In-Chang Hwang, Eun Ju Chun.
  - **Methodology:** In-Chang Hwang, Eun Ju Chun, Pan Ki Kim, Byoung Wook Choi.
  - **Project administration:** In-Chang Hwang, Eun Ju Chun.
  - **Software:** In-Chang Hwang, Eun Ju Chun, Pan Ki Kim, Byoung Wook Choi.
  - **Supervision:** In-Chang Hwang, Eun Ju Chun.
  - **Validation:** In-Chang Hwang, Eun Ju Chun.
  - **Visualization:** In-Chang Hwang, Eun Ju Chun, Pan Ki Kim, Byoung Wook Choi.
  - **Writing – original draft:** In-Chang Hwang, Eun Ju Chun.
  - **Writing – review & editing:** In-Chang Hwang, Eun Ju Chun, Myeongju Kim, Jiesuck Park, Hong-Mi Choi, Yeonyee E. Yoon, Goo-Yeong Cho.

**Item #4**

Thank you for stating the following in the Competing Interests section: 
*Pan Ki Kim and Byoung Wook Choi are founders of Phantomics, Inc. (Seoul, Korea), which supports the software used in this study; however, they did not participate in the data analysis. The other authors declare no conflicts of interest.*
We note that one or more of the authors are employed by a commercial company: Phantomics, Inc. (Seoul, Korea)

**a.** Please provide an amended Funding Statement declaring this commercial affiliation, as well as a statement regarding the Role of Funders in your study. If the funding organization did not play a role in the study design, data collection and analysis, decision to publish, or preparation of the manuscript and only provided financial support in the form of authors' salaries and/or research materials, please review your statements relating to the author contributions, and ensure you have specifically and accurately indicated the role(s) that these authors had in your study. You can update author roles in the Author Contributions section of the online submission form.

Please also include the following statement within your amended Funding Statement. 
“The funder provided support in the form of salaries for authors [insert relevant initials], but did not have any additional role in the study design, data collection and analysis, decision to publish, or preparation of the manuscript. The specific roles of these authors are articulated in the ‘author contributions’ section.”
If your commercial affiliation did play a role in your study, please state and explain this role within your updated Funding Statement.

**b.** Please also provide an updated Competing Interests Statement declaring this commercial affiliation along with any other relevant declarations relating to employment, consultancy, patents, products in development, or marketed products, etc.

Within your Competing Interests Statement, please confirm that this commercial affiliation does not alter your adherence to all PLOS ONE policies on sharing data and materials by including the following statement: "This does not alter our adherence to  PLOS ONE policies on sharing data and materials.” (as detailed online in our guide for authors <http://journals.plos.org/plosone/s/competing-interests>) . If this adherence statement is not accurate and  there are restrictions on sharing of data and/or materials, please state these. Please note that we cannot proceed with consideration of your article until this information has been declared.

Please include both an updated Funding Statement and Competing Interests Statement in your cover letter. We will change the online submission form on your behalf.

**Response to Item #4**

We sincerely thank the editor for this considerate comment. The Funding Statement, Competing Interests Statement, and Author Contributions Statement were revised, as provided for Item #3 above.

**Item #5**

Please review your reference list to ensure that it is complete and correct. If you have cited papers that have been retracted, please include the rationale for doing so in the manuscript text, or remove these references and replace them with relevant current references. Any changes to the reference list should be mentioned in the rebuttal letter that accompanies your revised manuscript. If you need to cite a retracted article, indicate the article’s retracted status in the References list and also include a citation and full reference for the retraction notice.

**Response to Item #5**

We reviewed our reference list, and confirm that it is complete and correct.
